# LOCAL FUNDING FOR SOCIAL CARE FOR OLDER COMMUNITY MEMBERS: A BOTTOM-UP STRATEGY THAT IS ALSO GOOD POLITICS

**DOI:** 10.1093/geroni/igae098.0308

**Published:** 2024-12-31

**Authors:** Robert Applebaum, Bailee Brekke

**Affiliations:** Miami University, Oxford, Ohio, United States; Miami University, W. College Corner, Indiana, United States

## Abstract

The national response to providing home and community-based services for older people with disability relies almost exclusively on the Medicaid program. Because 90% of older community members are not eligible for Medicaid this means that until a crisis the vast majority of older individuals are not eligible for public support. In 15 states across the nation, communities have raised local funds to support social care services to assist in maintaining community independence. Raising more than $400 million, primarily through property tax levies, the more than 400 communities using this approach fund an array of social care services, such as personal care, home modification, meals, and transportation targeted toward a population above the Medicaid eligibility threshold. This strategy is particularly salient in Ohio, where 74 or the state’s 88 counties have levies totaling more than $200 million. This presentation will describe both the programs and politics associated with this funding approach. The presentation will highlight how funds are use and the target population served. Local funding has been successful at the polls, and a review of voting patterns found a more than 95% passage rate in the Ohio communities, with no differences in rates between red and blue counties of the state.

